# Clinical characteristics and treatment outcomes of patients with macrolide-resistant *Mycobacterium avium* complex pulmonary disease: a systematic review and meta-analysis

**DOI:** 10.1186/s12931-019-1258-9

**Published:** 2019-12-18

**Authors:** Youngmok Park, Eun Hye Lee, Inkyung Jung, Goeun Park, Young Ae Kang

**Affiliations:** 1 Division of Pulmonology, Department of Internal Medicine, Severance Hospital, Yonsei University College of Medicine, 50-1 Yonsei-ro, Seodaemun-Gu, 03722 Seoul, Republic of Korea; 2 Division of Biostatistics, Department of Biomedical Systems Informatics, Yonsei University College of Medicine, Seoul, Republic of Korea

**Keywords:** *Mycobacterium avium* complex, *Mycobacterium avium-intracellulare* infection, Macrolides, Drug resistance, Clarithromycin

## Abstract

**Background:**

Macrolide is a key drug in the treatment of *Mycobacterium avium* complex pulmonary disease (MAC-PD). Macrolide-resistant MAC is gaining importance, but there are little data in clinical characteristics and treatment outcomes of macrolide-resistant MAC-PD (MR-MAC-PD).

**Methods:**

We performed a systematic review and meta-analysis of published studies reporting clinical characteristics and treatment outcomes of patients with MR-MAC-PD. Risk of bias was assessed using the modified Newcastle-Ottawa Scale.

**Results:**

Nine studies (seven retrospective and two prospective) comprising 319 patients were identified through a database search. Around 73% were women, and 52% had the fibrocavitary form. Pooled sputum culture conversion rate after combined multiple antibiotics or surgical resection was 21% (95% confidence interval [CI], 14–30%), and the one-year all-cause mortality was 10% (95% CI, 5–20%). There was no significant difference in treatment outcomes between nodular bronchiectatic and fibrocavitary types.

**Conclusions:**

Even combination therapy with fluoroquinolone, aminoglycoside, and surgical resection, the treatment outcomes of MR-MAC-PD were poor. The investigation of new treatment modalities is urgent.

## Background

The incidence and prevalence of nontuberculous mycobacterial (NTM) pulmonary disease are increasing worldwide [1–3]. *Mycobacterium avium* complex (MAC), mainly comprising *M. avium* and *M. intracellulare*, has been reported as the most common etiology of NTM pulmonary disease in many countries such as North America and East Asia [3].

Macrolides, such as clarithromycin and azithromycin, are indispensable to the antibiotic treatment of MAC pulmonary disease (MAC-PD). A macrolide-based multidrug regimen comprising ethambutol and rifamycin has been recommended as the first-line therapy for patients with MAC-PD [1, 2, 4]. However, the development of macrolide resistance indicated poor treatment outcomes and increased mortality [5–8], similar to the prognosis of multidrug-resistant tuberculosis (MDR-TB) [7].

Only a few studies with a limited number of patients have evaluated the clinical characteristics and treatment outcomes of macrolide-resistant MAC-PD (MR-MAC-PD), and the results were inconsistent in terms of risk factors and optimal treatment modalities. The different definitions of treatment outcomes in MAC-PD also led to inconsistency in treatment success. Conducting a prospective controlled study with a large number of patients is challenging because the development of macrolide resistance is unpredictable, and there is no evidence-based treatment regimen in MR-MAC-PD. Therefore, in the present study, we aimed to understand the clinical characteristics and treatment outcomes of MR-MAC-PD through a systematic review and meta-analysis.

## Methods

This study was performed according to the Preferred Reporting Items for Systematic Reviews and Meta-Analyses guidelines [9]. The protocol was registered on PROSPERO (registration number: CRD42019118499).

### Search strategies

We searched Medline, Embase, Cochrane library, and ProQuest databases to identify full-length articles published up to August 25, 2019. The search strategy for each database is presented in Additional file 2: Table S1, S2, S3, and S4. Duplicates, as well as case reports, reviews, conference abstracts, newspaper articles, nonclinical studies, and animal studies were excluded. English studies were selected.

### Eligibility criteria

The inclusion criterion was original studies on MR-MAC-PD patients without human immunodeficiency virus (HIV) infection. Relevant studies were independently selected by two reviewers (Y Park and EH Lee). The authors initially screened the articles by title and abstract, and then assessed the full text as needed. Studies with less than 5 patients were excluded. No restrictions were applied regarding study design or methods of data collection (prospective or retrospective).

### Data extraction and quality assessment

Two coding authors (Y Park and EH Lee) extracted data from the selected publications with a pre-defined data extraction form. The following information was recorded: study characteristics (authors, setting, study design, criteria for macrolide resistance), patient characteristics (age, sex, radiologic types, etc.), and treatment outcomes (sputum culture conversion and the one-year all-cause mortality rate). Study quality was assessed using a modified Newcastle-Ottawa Scale [10]. The one-year all-cause mortality rate provided with Kaplan-Meier curve was obtained by digitizing the figure using the online software Web Plot Digitizer [11].

### Statistical analysis

Pooled estimates and 95% CI were calculated using either fixed-effects or random-effects model. Heterogeneity was quantified in terms of Q- and I^2^-statistics. If a significant heterogeneity was present (*P*-values for Q-statistics < 0.10) [12], pooled estimates from random-effects models were reported. Publication bias was assessed using a funnel plot. We used R (v. 3.6.0) in all statistical analyses.

## Results

### Study selection and identified studies

**Fig. 1:**
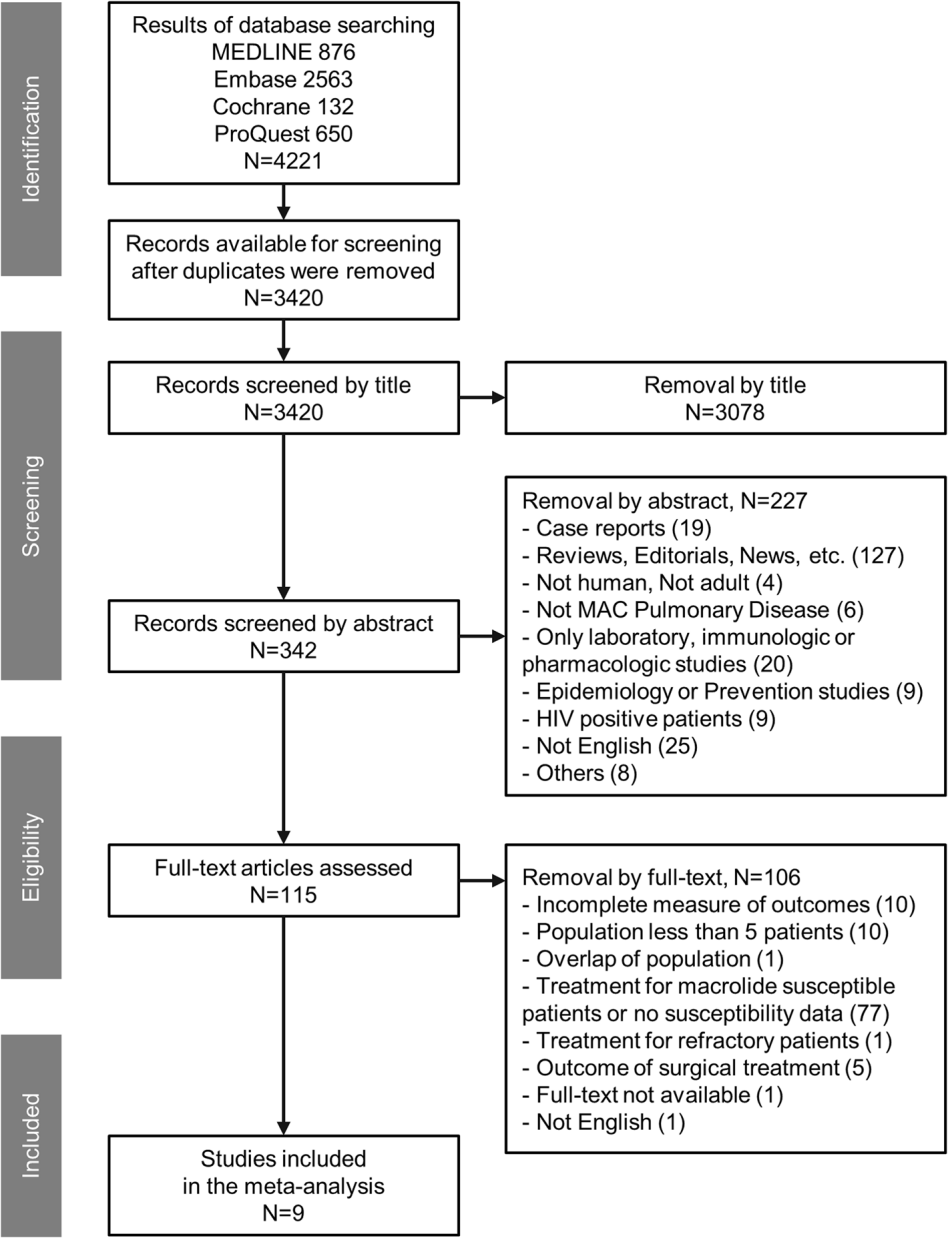
Flowchart describing the selection of studies included in the analysis. Abbreviations: MAC, *M. avium* complex pulmonary disease; HIV, human immunodeficiency virus

A total of 4221 studies were identified from the database search. Among them, 3420 publications were selected after comparison of results and de-duplication. The selected studies were screened by title, abstract, and full text. Figure 1 shows the selection process and exclusion criteria. Finally, we included nine studies on MR-MAC-PD in this meta-analysis [5–8, 13–17].

The identified studies and their key characteristics are listed in Table 1. There were two prospective studies, and the other seven studies were of retrospective descriptive design. A total of 319 patients were eligible for the analysis. In eight studies, MAC isolates with a clarithromycin minimal inhibitory concentration ≥ 32 μg/mL in the broth microdilution method were defined as macrolide-resistant.

**Table 1:** Summary of the identified studies

Study	Study design	Country	Enrollment	Inclusion criteria*	MR-MAC-PD Pts /Total Pts, n / n (%)	Age, year	Female, n (%)	BMI, kg/m^2^	FC type, n (%)	Never smoker, n (%)	*M. intracellulare / M. avium,* n (%) */* n (%)
Tanaka 1999 [13]	Prospective observational	Japan	Nov 1992 – Nov 1997	> 32	6 / 46 (13)	NA	4 (67)	NR	NA	NA	NA
Griffith 2006 [5]	Retrospective observational	United States	1991–2005	≥ 32	51 / 51 (100)	64.7 ± 13.6	28 (55)	NR	27 (53)	18 (35)	41 (77) / 12 (23)
Moon 2016 [6]	Retrospective observational	South Korea	Jan 2002 – Dec 2014	≥ 32	34 / 34 (100)	65 (61–70)	11 (32)	19.7 (17.3–21.2)	19 (56)	20 (59)	21 (62) / 13 (38)
Morimoto 2016 [7]	Retrospective observational	Japan	Sep 2005 – Jul 2014	> 32	90 / 90 (100)	68 (60–74)	67 (74)	17.4 (15.7–19.6)	27 (30)	67 (74)	15 (17) / 50 (56)
Kadota 2016 [8]	Retrospective observational	Japan	Jan 2009 – Jun 2013	≥ 32	33 / 33 (100)	67 ± 9	31 (94)	17.2 ± 5.0	25 (76)	30 (91)	NR
Yagi 2017 [14]	Retrospective observational	Japan	Jan 2014 – May 2016	≥ 32	9 / 26 (35)	NA	8 (89)	NA	4 (44)	NA	0 (0) / 9 (100)
Aznar 2018 [15]	Retrospective observational	Canada	Jul 2003 – Dec 2016	NR	8 / 54 (15)	surgery 67.5 (64–69.5) non-surgery 66 (57.5–77.5)	8 (100)	NA	0 (0)	NR	NA
Griffith 2018 [16]	Prospective randomized	Global^†^	May 2015 – Jan 2017	≥ 32	73 / 336 (22)	NA	NA	NA	NR	NA	NR
Asakura 2019 [17]	Retrospective observational	Japan	Jan 2010 – Jul 2017	≥ 32	15 / 31 (48)	NA	12 (80)	≤18.5: 9 (60%) > 18.5: 6 (40%)	NA	NA	NA

**Note:** Data are presented as mean ± standard deviation or median (interquartile range) unless otherwise indicated

*Minimal inhibitory concentration of clarithromycin, μg/mL

†The study was conducted at 127 clinical centers in 18 countries in North America, Asia-Pacific region, and Europe

Abbreviations: MR-MAC-PD macrolide-resistant *M. avium* complex pulmonary disease, BMI body mass index, FC fibrocavitary, Pts patients, NR not reported in the article, NA specific data for MR-MAC-PD patients are not available

### Clinical characteristics

**Fig. 2:**
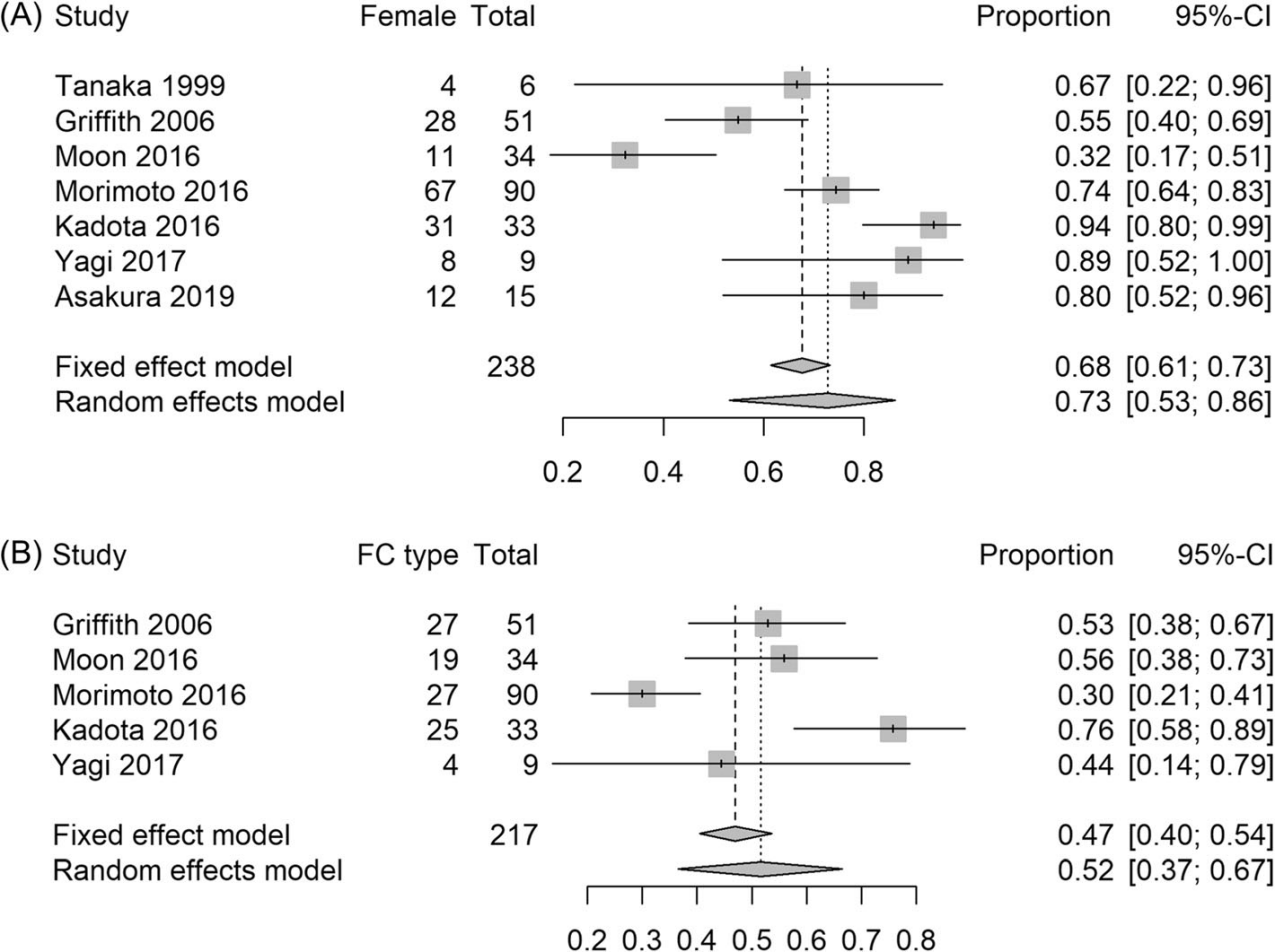
Clinical characteristics of patients with macrolide-resistant *M. avium* complex pulmonary disease. Forest plots for (**a**) female proportion and (**b**) fibrocavitary (FC) type disease proportion. Note: A study by Aznar et al. [15] was excluded from this analysis because it is designed to match sex and radiologic types

The mean age of the patients ranged from 65 to 68 years, and the proportion of women ranged from 32 to 100% (Table 1). The pooled estimate for the proportion of women was 73% (95% Confidence interval [CI], 53–86%, Fig. 2a) with random-effects model; I^2^ statistic was 84%, and Q-statistic was 31.2 (*P* < 0.001), indicating a high level of heterogeneity.

Five studies reported the percentage of fibrocavitary (FC) type of the disease. The pooled estimate was 52% (95% CI, 37–67%, Fig. 2B) with random-effects model; the I^2^ statistic was 76%, and Q-statistic was 21.4 (P < 0.001), representing substantial heterogeneity. Four studies [5–7, 14] reported the proportion of *M. avium* and *M. intracellulare* separately, and the percentage of *M. avium* ranged from 23 to 100% (Table 1).

As we excluded the studies with HIV infected subjects, most patients had chronic lung diseases such as asthma, chronic obstructive pulmonary disease, bronchiectasis, chronic pulmonary aspergillosis, or history of pulmonary tuberculosis.

### Sputum culture conversion rate and the one-year all-cause mortality rate

**Fig. 3:**
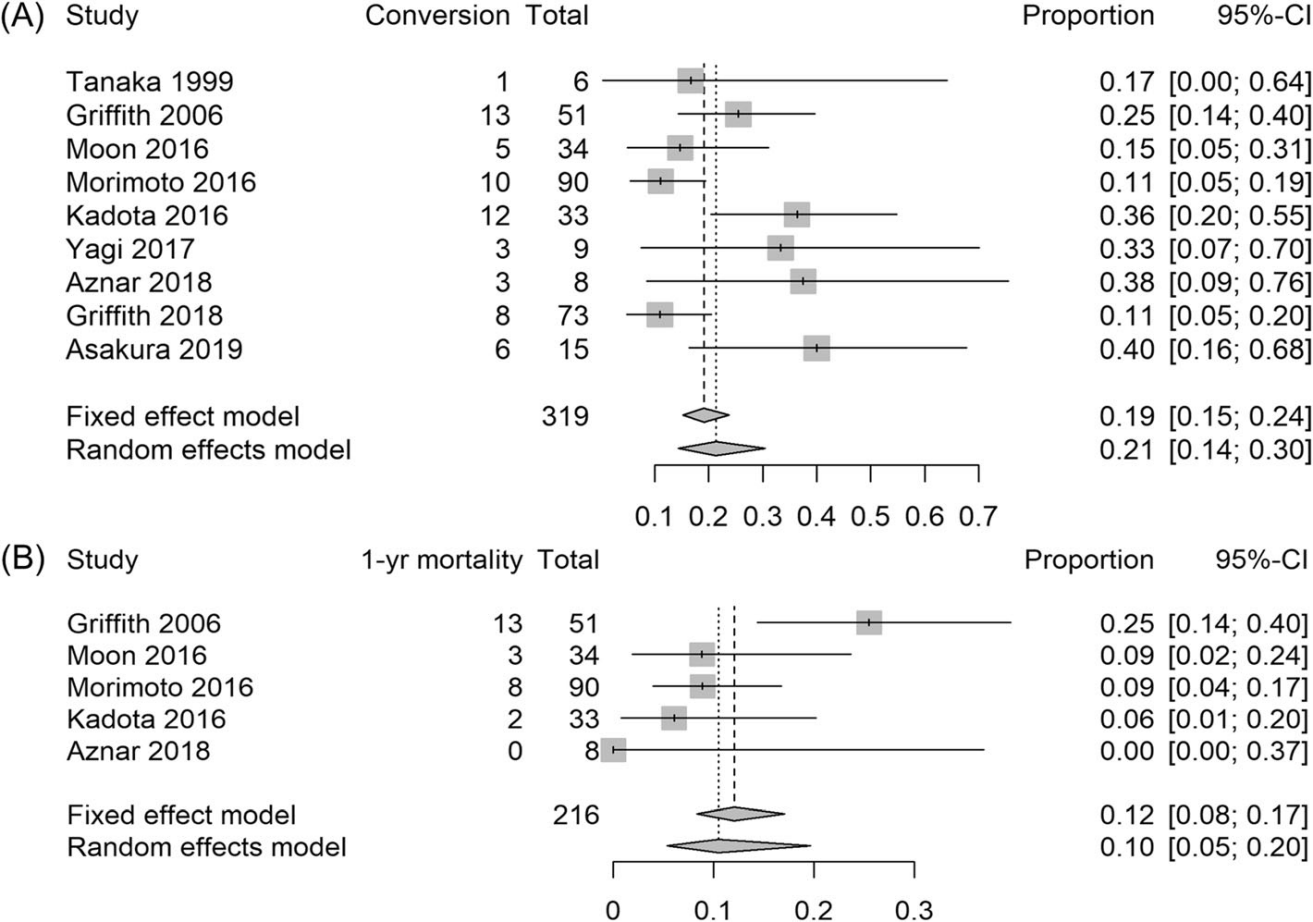
Treatment outcomes of patients with macrolide-resistant *M. avium* complex pulmonary disease. Forest plots for (**a**) sputum culture conversion rate and (**b**) one-year all-cause mortality rate

The definition of sputum culture conversion in each study is described in Table 2. The reported sputum culture conversion rate ranged from 11% [7, 16] to 38% [15], and the overall pooled estimate with random-effects model was 21% (95% CI, 14–30%, Fig. 3a). The I^2^ statistic was 57%, and the Q-statistic was 20.3 (*P* = 0.009).

**Table 2:** Treatment regimen and outcomes of MR-MAC-PD

Study	N	Treatment regimen, n (%)	FQ, n (%)	AG, n (%)	CFZ, n(%)	Surgery, n (%)	Treatment duration, months
Tanaka 1999 [13]	6	NA	NA	NA	NR	NR	NA
Griffith 2006 [5]	51	Surgery, prolonged (≥6 mo) AG: 14 (27) Surgery, no prolonged AG: 2 (4) No surgery, prolonged AG: 8 (16) No surgery, no prolonged AG: 27 (53)	CIPX 4 (8) GTFX 6 (12)	SM 24 (47) AMK 11 (22)	4 (8)	16 (31)	NA*
Moon 2016 [6]	34	Macrolide 16 (47), EMB 25 (74), RIF or RFB 34 (100)	MFX 17 (50)	SM 13 (38)	4 (12)	2 (6)	23.0 (16.8–45.3)
Morimoto 2016 [7]	90	CAM 55 (61), RFB 15 (17) FQ 56 (62) for median 12 mo AG 52 (58) for median 6 mo	STFX 44 (49) MFX 11 (12) LFX 8 (9)	AMKi 32 (36) AMKn 7 (8) KM 10 (11) SM 4 (4)	NR	11 (12)	21 (10–37)
Kadota 2016 [8]	33	CAM 24 (73), AZM 2 (6), Regimen without macrolide 7 (21) FQ for median 5 mo	LFX 16 (48) MFX 2 (6) STFX 2 (6)	KM 9 (27)	NR	0 (0)	10.4 ± 1.6^¶^
Yagi 2017 [14]	9	RIF + EMB + FQ + AMKn: 4 (44) RIF (RFB) + FQ + AMKn: 2 (22) EMB + FQ + AMKn: 1 (11) RIF + EMB + AMKn: 1 (11) CAM + EMB + AMKn: 1 (11)	STFX 6 (67) MFX 1 (11)	AMKn 9 (100)	0 (0)	0 (0)	(6–16)^#^
Aznar 2018 [15]	8	All patients received GBT, including a macrolide, ethambutol, and a rifamycin when possible. Four patients had adjuvant surgery.	NA	NA	NA	4 (50)	> 12
Griffith 2018 [16]	73	Patients were randomly assigned in a 2:1 ratio to receive ALIS to GBT or GBT alone.	NA	ALIS 51 (70)	NA	NA	NA
Asakura 2019 [17]	15	GBT plus STFX 200 mg 14 (93) GBT plus STFX 100 mg 1 (7)	STFX 15 (100)	AMKi or AMKn 5 (33)	NR	2 (13)	≥12 mo: 13 (87%) < 12 mo: 2 (13%)
Study	N	Sputum culture conversion definition	Conversion, n (%)	Mortality, n (%)	Follow-up duration, months		
Tanaka 1999 [13]	6	Consecutive negative cultures during a 3-month period	1 (17)	NR	NA		
Griffith 2006 [5]	51	A minimum of three consecutive negative cultures over a minimum time of three months	13 (26)	1-yr 13 (25) 2-yr 17 (33)	(16–84)^†^ (18–54)^‡^		
Moon 2016 [6]	34	Three consecutive negative cultures, 2- to 3-month intervals	5 (15)^§^	1-yr 3 (9) 3-yr 8 (24) 5-yr 16 (47)	39.3 (22.9–43.4)		
Morimoto 2016 [7]	90	Three consecutive negative cultures	10 (11)	1-yr 8 (9) 2-yr 13 (15) 3-yr 15 (17) 5-yr 26 (29)	21 (10–37)		
Kadota 2016 [8]	33	Two consecutive negative cultures. If the patient could not expectorate sputum, it was considered to have converted to negative	12 (36)	1-yr 2 (6)	10.4 ± 1.6^¶^		
Yagi 2017 [14]	9	Three consecutive negative cultures after amikacin inhalation	3 (33)	NR	NR		
Aznar 2018 [15]	8	Persistently negative cultures or when unable to provide sputum specimens for culture during at least 12 months	3 (38)	1-yr 0 (0)	> 12		
Griffith 2018 [16]	73	Three consecutive monthly MAC-negative cultures by Month 6	8 (11)	NR	6**		
Asakura 2019 [17]	15	Three consecutive negative cultures. If the patients did not expectorate sputum, the status was recorded as negative.	6 (40)	NA	NA		

**Note:** Data are presented as mean ± standard deviation or median (interquartile range) unless otherwise indicated

*Duration of prolonged aminoglycoside therapy for 14 patients is 12.0 ± 4.1 (range 7–19) months

†Range of follow-up duration for survived patients who remained culture-positive after resistance diagnosis

‡Range of follow-up duration for survived patients who were cured or culture converted after resistance diagnosis

§Number of patients who achieved ‘favorable outcome,’ which was defined as sputum culture conversion within 12 months after initiation of treatment and maintenance of a negative culture for 12 months or longer on treatment

#Range of AMKn duration except for one patient who discontinued within 1 month due to pneumothorax. Two of eight patients used AMKi at some interval

** The study reported primary results at 6 months of ongoing phase 3 CONVERT study

Abbreviations: MR-MAC-PD macrolide-resistant *M. avium* complex pulmonary disease, CAM clarithromycin, AZM azithromycin, EMB ethambutol, RIF rifampicin, RFB rifabutin, CIPX ciprofloxacin, LFX levofloxacin, MFX moxifloxacin, STFX sitafloxacin, GTFX gatifloxacin, AMKn amikacin inhalation, AMKi amikacin injection, ALIS amikacin liposome inhalation suspension, SM streptomycin, KM kanamycin, CFZ clofazimine, GBT guideline-based therapy, NR not reported in the article, NA specific data for MR-MAC-PD patients are not available

The one-year all-cause mortality rate was reported in four studies (Fig. 3b). It ranged from 0 to 25% [5], and the pooled estimate was 10% (95% CI, 5–20%) with I^2^ statistic of 53% and Q-statistic of 9.60 (*P* = 0.048).

We conducted a subgroup analysis by radiologic types of MAC-PD. Four studies reported the sputum culture conversion rate between nodular bronchiectatic (NB) and FC types. The estimated odds ratio for sputum culture conversion in NB type compared with the FC type was 0.56 (95% CI, 0.24–1.30, Additional file 1: Fig. S1A).

Three studies reported the odds ratios for the one-year all-cause mortality rate in NB to FC type: 2.20 (95% CI, 0.61–7.99) [5]; 0.15 (95% CI, 0.01–3.19) [6]; and 0.06 (95% CI, 0.00–1.23) [7]. The pooled estimate was 0.38 (95% CI, 0.03–4.30) with random-effects model (Additional file 1: Fig. S1B); the interpretation needs caution owing to the limited number of studies.

### Treatment modalities of MR-MAC-PD

According to the studies of Tanaka et al. [13] and Griffith et al. [5], more than half of the patients with MR-MAC-PD were treated with macrolide monotherapy or a two-drug regimen comprising macrolides before the detection of macrolide resistance. In contrast, patients treated with standard multidrug regimen before the development of macrolide resistance were also substantial in the studies of Moon et al. (65%) and Morimoto et al. (28%) [6, 7].

Treatment regimens after the detection of macrolide resistance in each study are presented in Table 2. For the treatment of MR-MAC-PD, new treatment perspectives are tried, such as clofazimine, amikacin inhalation, amikacin liposomal inhalation suspension (ALIS), and surgery.

### Assessment of methodological quality

When methodological quality was assessed using the modified Newcastle-Ottawa Scale, most studies had a low risk of bias for measurement of macrolide resistance, and low to moderate risk of bias in the patient selection and assessment of treatment outcomes (Additional file 2: Table S5).

## Discussion

Newer macrolides, including clarithromycin and azithromycin, are cornerstones in the antibiotic treatment of MAC-PD, and the development of macrolide resistance is associated with poor treatment outcomes [4]. However, there are limited studies on treatment modalities and outcomes for MR-MAC-PD. In this study, we aimed to understand the clinical characteristics and treatment outcomes of MR-MAC-PD through a systematic review and meta-analysis, and found that the overall sputum culture conversion rate was 21% (95% CI, 14–30%), and the one-year all-cause mortality rate was 10% (95% CI, 5–20%). There were no differences in sputum culture conversion rate and the one-year all-cause mortality rate between NB and FC type of MR-MAC-PD.

The treatment of MAC-PD is complicated, and the treatment results are not satisfactory. Even in macrolide-susceptible MAC-PD, the treatment success rate with macrolide containing multidrug regimens ranged from 60% [18] to 65.7% [19]. Sustained sputum culture conversion rate with macrolide-free regimens was 38% in a previous systematic review [20]. In our present study, the pooled estimate of sputum culture conversion rate was 21% (95% CI, 14–30%), which represents a worse treatment outcome of MR-MAC-PD. Pan et al. reported that microbiologic persistence in patients with MAC-PD could lead to an increased risk of radiographic progression [21]. In the same perspective, low sputum culture conversion rate in MR-MAC-PD could be a predictor of disease progression including radiographic progression.

Patients with MAC-PD are at a significant risk of death. Diel et al. [22] reported a pooled estimate of the five-year all-cause mortality rate of patients with MAC-PD as 27% (95% CI, 21–33%). Moon et al. [6] and Morimoto et al. [7] reported five-year all-cause mortality rates of MR-MAC-PD as 47 and 29%, respectively. The pooled estimate of the one-year mortality rate was 10% (95% CI, 5–20%) in this study. Several studies have demonstrated the incremental impact of NTM infection on mortality [23–25]; however, the assessment of NTM-related mortality is difficult because the proportion of deaths attributed to NTM infection depends mostly on how clinicians decide the cause of death. In addition, there are limited data on the mortality of MR-MAC-PD compared to the general population and patients with macrolide-susceptible MAC-PD. Considering the chronic features of NTM infection, long-term mortality assessment is necessary for patients with MR-MAC-PD.

Morimoto et al. [7] compared the treatment outcome of MR-MAC-PD to that of 311 patients with MDR-TB; the 5-year survival rates between the two groups were similar (71% vs. 75%, *P* = 0.6). MDR-TB is considered a severe health concern worldwide. The World Health Organization reported that only 55% of patients with MDR-TB in 2015 successfully completed medication; the treatment failed in 8% of the patients, and 15% died [26].

There are a few explanations for the emergence of macrolide resistance in MAC-PD. First, inappropriate regimen as first-line treatment may trigger macrolide resistance. Griffith et al. [5] reported that the majority (76%) of patients with MR-MAC-PD started their initial treatment with macrolide monotherapy or the combination of a macrolide and a fluoroquinolone. Morimoto et al. [7] showed that 60.2% of patients did not receive proper multidrug regimens, such as clarithromycin monotherapy, clarithromycin plus fluoroquinolone, and regimens without ethambutol. Moon et al. [6] also reported that one-third of the population did not receive ethambutol owing to its adverse events.

Second, relatively low concentrations of core drugs and high bacterial burden have been suggested as an explanation for macrolide resistance. Concomitant use of rifamycin is related to reduced serum levels of macrolide, particularly clarithromycin [27, 28]. Moon et al. [6] and Kadota et al. [8] reported that macrolide resistance can occur even when patients were treated with proper multidrug regimen, because a small proportion of patients received macrolide monotherapy (32% in the study of Moon et al. and 18% in the study of Kadota et al., respectively) or a two-drug combination with a macrolide (33% in the study of Moon et al. and 12% in the study of Kadota et al., respectively).

There is no proven treatment modality for MR-MAC-PD yet. Maintenance of macrolide after the detection of macrolide resistance is frequent. Surgical intervention and prolonged parenteral aminoglycoside administration are the primary treatment strategies for the treatment of MR-MAC-PD (Table 2). The recent British Thoracic Society guidelines recommend adding another drug such as isoniazid, moxifloxacin, or nebulized amikacin for treatment of MR-MAC-PD. However, the efficacy of these treatment regimens remains inconclusive [2]. Recently, clofazimine, bedaquiline, and ALIS have been used for the treatment of refractory MAC-PD, including MR-MAC-PD [16, 29, 30]. In the CONVERT study [16], a prospective open-label, randomized study for the treatment of patients with refractory MAC-PD, ALIS with guideline-based therapy (GBT) showed culture conversion rate of 29.0% compared to 8.9% of GBT alone. Among MR-MAC-PD group, culture conversion was achieved by 13.7% of patients in the ALIS + GBT arm and 4.5% in the GBT-alone arm. Even this latest ALIS therapy, treatment outcome of MR-MAC-PD was poor. Bedaquiline was also recently tried as a treatment option for refractory NTM-PD, although the study was preliminary and the number of patients was small [29]. Therefore, for the treatment of MR-MAC-PD, new drugs or new pharmaceutical formulations of existing drugs should be investigated.

This is the first study to integrate the clinical characteristics and treatment outcomes of patients with MR-MAC-PD. Nevertheless, this study has several limitations. First, because only a small number of studies were enrolled in this analysis, we could not thoroughly evaluate the publication bias. Second, all the studies had no control group, and most of them were retrospective observational design. Therefore, we should interpret the results with caution. Third, the treatment outcomes were estimated from the various treatment modalities because there was no uniform protocol for the treatment of MR-MAC-PD. Fourth, we could not evaluate the long-term treatment outcomes of MR-MAC-PD owing to the relatively short follow-up duration of each study. In addition, we could not assess treatment outcomes including clinical and radiographic improvement.

In conclusion, the treatment outcomes of MR-MAC-PD were poor, the overall sputum culture conversion rate was 21% (95% CI, 14–30%), and the one-year all-cause mortality rate was 10% (95% CI, 5–20%). Despite the combination of multiple antibiotics including ALIS and surgical resection, the outcomes of MR-MAC-PD were poor The investigation of new treatment modalities is urgent for the treatment of MR-MAC-PD.

## Supplementary information

**Additional file 1: Fig. S1** Comparison of (A) sputum culture conversion rate and (B) one-year all-cause mortality rate between nodular bronchiectatic (NB) and fibrocavitary (FC) type disease of macrolide-resistant *M. avium* complex pulmonary disease

**Additional file 2: Table S1**. Database search strategy for MEDLINE. **Table S2** Database search strategy for Embase. **Table S3** Database search strategy for Cochrane library. **Table S4** Database search strategy for ProQuest. **Table S5** Quality assessment of included studies using a modified Newcastle-Ottawa scale

## Data Availability

This study was a re-analysis of existing data, which is openly available at locations cited in the reference section.

## Abbreviations

ALIS: Amikacin liposomal inhalation suspension
CI: Confidence interval
FC: Fibrocavitary
GBT: Guideline-based therapy
HIV: Human immunodeficiency virus
MAC: *Mycobacterium avium* complex
MAC-PD: *Mycobacterium avium* complex pulmonary disease
MDR-TB: Multidrug-resistant tuberculosis
MR-MAC-PD: Macrolide-resistant *Mycobacterium avium* complex pulmonary disease
NB: Nodular bronchiectatic
NTM: Nontuberculous mycobacteria
