# Supplementary figures and images for: Clinical characteristics and treatment outcomes of patients with macrolide-resistant Mycobacterium avium complex pulmonary disease: a systematic review and meta-analysis
Source: Respir Res. 2019 Dec 18;20:286. doi: 10.1186/s12931-019-1258-9 (PMC6921583; doi:10.1186/s12931-019-1258-9)

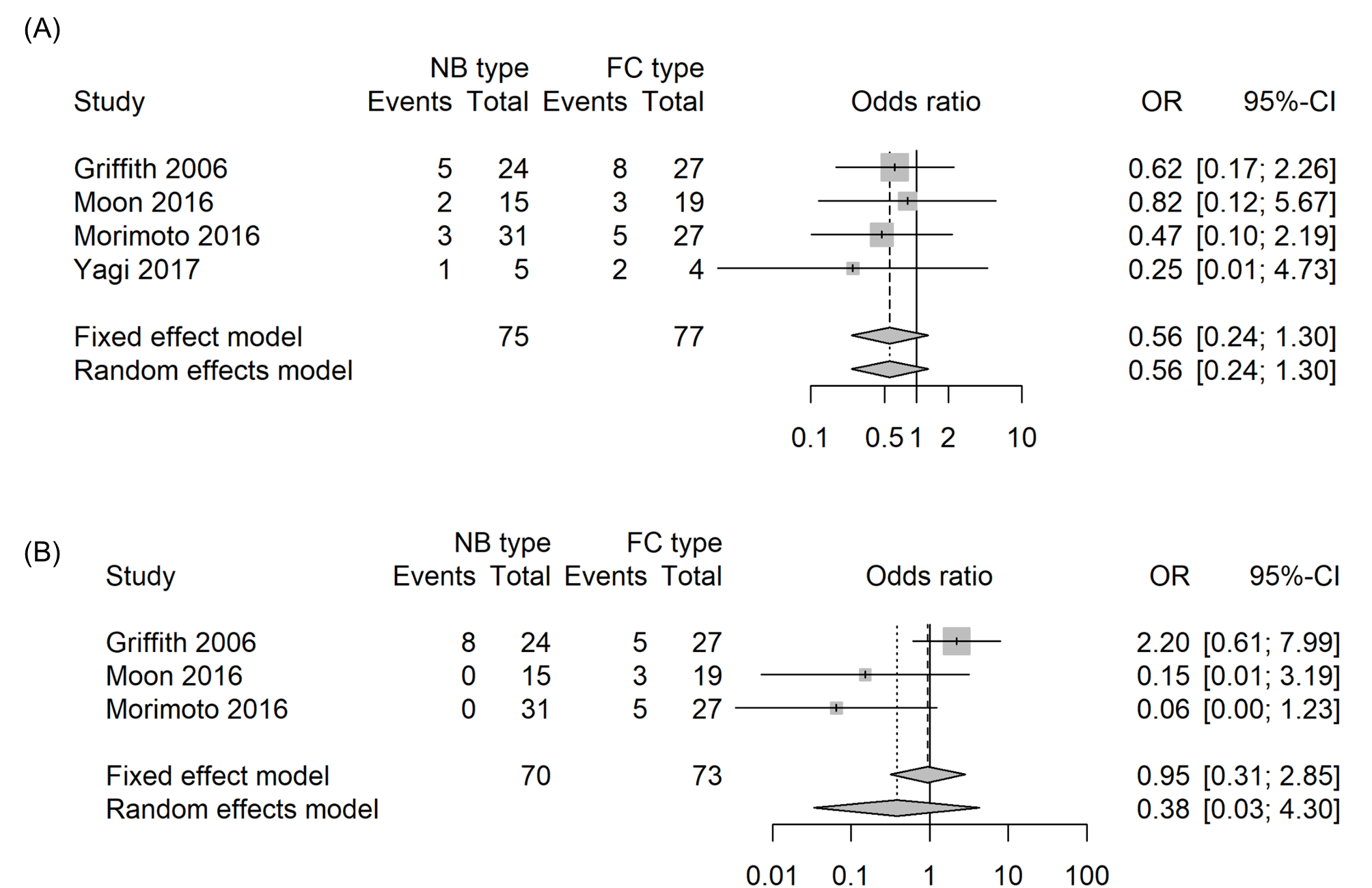

Supplement: Supplementary file 1 — Additional file 1: Fig. S1 Comparison of (A) sputum culture conversion rate and (B) one-year all-cause mortality rate between nodular bronchiectatic (NB) and fibrocavitary (FC) type disease of macrolide-resistant M. avium complex pulmonary disease [file 12931_2019_1258_MOESM1_ESM.tif]
