# Supplementary material for: Clinical characteristics and treatment outcomes of patients with macrolide-resistant Mycobacterium avium complex pulmonary disease: a systematic review and meta-analysis
Source: Respir Res. 2019 Dec 18;20:286. doi: 10.1186/s12931-019-1258-9 (PMC6921583; doi:10.1186/s12931-019-1258-9)
Supplement: Supplementary file 2 — Additional file 2: Table S1. Database search strategy for MEDLINE. Table S2 Database search strategy for Embase. Table S3 Database search strategy for Cochrane library. Table S4 Database search strategy for ProQuest. Table S5 Quality assessment of included studies using a modified Newcastle-Ottawa scale [file 12931_2019_1258_MOESM2_ESM.docx]

**Table S1** Database search strategy for MEDLINE

| \| Search \| Query \| Results \| \| --- \| --- \| --- \| \| #8 \| Search (#5) AND #6 Filters: Humans \| 876 \| \| #7 \| Search (#5) AND #6 \| 1051 \| \| #6 \| Search (#3) OR #4 \| 117995 \| \| #5 \| Search (#1) OR #2 \| 12322 \| \| #4 \| Search ((macrolides[MeSH Terms]) OR clarithromycin[MeSH Terms]) OR azithromycin[MeSH Terms] \| 106261 \| \| #3 \| Search ((macrolide) OR clarithromycin) OR azithromycin \| 117995 \| \| #2 \| Search ((mycobacterium avium-intracellulare infection[MeSH Terms]) OR mycobacterium avium complex[MeSH Terms]) OR mycobacterium avium[MeSH Terms] \| 8695 \| \| #1 \| Search ((mycobacterium avium complex) OR mycobacterium avium) OR mycobacterium intracellulare \| 12322 \| |  |
| --- | --- | --- | --- | --- | --- | --- | --- | --- | --- | --- | --- | --- | --- | --- | --- | --- | --- | --- | --- | --- | --- | --- | --- | --- | --- | --- | --- | --- |

**Table S2** Database search strategy for Embase

| Search | Query | Results |
| --- | --- | --- |
| #8 | #7 AND [humans]/lim | 2563 |
| #7 | #5 AND #6 | 2961 |
| #6 | #3 OR #4 | 298936 |
| #5 | #1 OR #2 | 16475 |
| #4 | 'macrolide'/exp OR 'clarithromycin'/exp OR 'azithromycin'/exp | 296001 |
| #3 | macrolide OR clarithromycin OR azithromycin | 87161 |
| #2 | 'mycobacterium avium complex'/exp OR 'mycobacterium avium'/exp OR 'mycobacterium intracellulare'/exp | 13615 |
| #1 | mycobacterium AND avium AND complex OR (mycobacterium AND avium) OR (mycobacterium AND intracellulare) | 16305 |

**Table S3** Database search strategy for Cochrane library

| Search | Query | Results |
| --- | --- | --- |
| #1 | (mycobacterium avium complex) OR (mycobacterium avium) OR (mycobacterium intracellulare) (Word variations have been searched) | 310 |
| #2 | MeSH descriptor: [Mycobacterium avium-intracellulare Infection] explode all trees | 116 |
| #3 | MeSH descriptor: [Mycobacterium avium Complex] explode all trees | 46 |
| #4 | MeSH descriptor: [Mycobacterium avium] explode all trees | 11 |
| #5 | #1 OR #2 OR #3 OR #4 | 272 |
| #6 | macrolide OR clarithromycin OR azithromycin | 5884 |
| #7 | MeSH descriptor: [Macrolides] explode all trees | 8148 |
| #8 | MeSH descriptor: [Clarithromycin] explode all trees | 1377 |
| #9 | MeSH descriptor: [Azithromycin] explode all trees | 872 |
| #10 | #6 OR #7 OR #8 OR #9 | 11663 |
| #11 | #5 AND #10 | 132 |

**Table S4** Database search strategy for ProQuest

| Set# | | Searched for | Results |
| --- | --- | --- | --- |
| S1 | (mycobacterium avium complex) OR (mycobacterium avium) OR (mycobacterium intracellulare) | | 4719 |
| S2 | macrolide OR clarithromycin OR azithromycin | | 11289 |
| S3 | S1 AND S2 | | 650 |

**Table S5** Quality assessment of included studies using a modified Newcastle-Ottawa scale

|  | Selection | | | Measurement | | Outcome | | |
| --- | --- | --- | --- | --- | --- | --- | --- | --- |
| Study | Adequate definition of cases | Representativeness of cases | Risk of bias | Macrolide-resistance confirmed through DST | Risk of bias | Assessment of outcome* | Adequacy of follow-up length† | Risk of bias |
| Tanaka 1999 | + | – | moderate | + | low | + | NA | moderate |
| Griffith 2006 | + | + | low | + | low | ++ | + | low |
| Moon 2016 | + | + | low | + | low | ++ | + | low |
| Morimoto 2016 | + | + | low | + | low | ++ | + | low |
| Kadota 2016 | + | + | low | + | low | ++ | – | moderate |
| Yagi 2017 | + | – | moderate | + | low | + | + | moderate |
| Aznar 2018 | + | – | moderate | – | high | ++ | + | low |
| Griffith 2018 | + | – | moderate | + | low | + | – | moderate |
| Asakura 2019 | + | – | moderate | + | low | ++ | + | low |

Note: ^*^Assessment for sputum culture conversion rate and mortality; ++ both, + either one, – none

^†^Follow-up duration more than 12 months; + yes, – no

Abbreviations: DST drug susceptibility testing, NR not reported in the article
